# Diversity of Bacteria and the Characteristics of Actinobacteria Community Structure in Badain Jaran Desert and Tengger Desert of China

**DOI:** 10.3389/fmicb.2018.01068

**Published:** 2018-05-23

**Authors:** Ye Sun, Yun-Lei Shi, Hao Wang, Tao Zhang, Li-Yan Yu, Henry Sun, Yu-Qin Zhang

**Affiliations:** ^1^Peking Union Medical College, Institute of Medicinal Biotechnology, Chinese Academy of Medical Sciences, Beijing, China; ^2^Desert Research Institute, Las Vegas, NV, United States

**Keywords:** *Actinobacteria*, diversity, microbial community, desert sands, 16S rRNA

## Abstract

To assess the diversity of actinobacterial taxa in desert sands and obtain the novel microbial resources, 79 and 50 samples were collected from the Badain Jaran (BJD) and Tengger Deserts (TGD) of China, respectively. High-throughput sequencing (HTS) of environmental 16S rRNA genes within these samples was conducted on an Illumina Miseq platform, using universal bacterial primers targeting the V3–V4 hypervariable region. Based on the HTS analyses, cultivation-dependent (CULD) techniques were optimized to identify the cultivable *Actinobacteria* members. A total of 346,766 16S rRNA gene reads comprising 3,365 operational taxonomic units (OTUs) were obtained from the BJD sands using HTS, while 170,583 reads comprising 1,250 OTUs were detected in the TGD sands. Taxonomic classification indicated that *Actinobacteria* was the predominant phylum, comprising 35.0 and 29.4% of the communities in BJD and TGD sands, respectively. Among the *Actinobacteria*, members of the *Geodermatophilaceae* were considerably abundant in both deserts, indicating that they represent ubiquitous populations within the deserts. At the genus level, *Arthrobacter* spp. and *Kocuria* spp. were dominant, and corresponded to 21.2 and 5.3% of the actinobacterial communities in BJD and TGD deserts, respectively. A total of 786 and 376 actinobacterial strains were isolated and identified from BJD and TGD samples, respectively. The isolates comprised 73 genera of 30 families within the phylum *Actinobacteria*. In addition to the *Geodermatophilaceae, Streptomyces* spp. were a prominent component of the isolates, comprising 25% of the isolates from BJD and 17.5% of those from TGD. Comparison of the actinobacterial community structure in other ecosystems indicated that *Geodermatophilaceae* was the main actinobacterial group in desert sands, which is consistent with our results. Additionally, in these desert habits, *Geodermatophilaceae* and some other core groups may promote or inhabit the subsequent members' occurrence or prosper to shape the bacteria community structure. However, it should be noted that a number of other low-abundance bacteria appear to be specific to desert sands, which are worth further investigation. In antimicrobial activity assays, 10.36 % of the tested isolates showed antimicrobial activities in one or more screens. Importantly, 37 of the newly isolated strains reported here represent novel taxa that could be valuable resources for further research of novel secondary metabolites and their ecological significance in deserts.

## Introduction

Extremophiles havex generated significant interest in the biological sciences in recent years, due to their unique genotypes, physiological functions, and secondary metabolites that hold great scientific and industrial value (Ciaramella, 1995). Deserts are extreme environments that are characterized by extreme aridity, intense solar UV radiation, and extreme shifts of temperature in day-night cycles. Consequently, deserts harbor numerous extremophiles (Subramani and Aalbersberg, 2013; Júlia et al., 2016). In addition to the above conditions, deserts feature chronic oligotrophy. Thus, sporadic vascular vegetation and microbiological crusts play critical roles in the primary production of this ecosystem, in addition to keeping soils from undergoing further desertification (Hawkes and Flechtner, 2002). Moreover, vegetation and microbiological crusts contribute greatly in shaping soil micro-ecosystem bacterial community structure in desert environments (Evans and Johansen, 1999; Belnap and Lange, 2003; Sun et al., 2015).

Deserts are typical of extreme, harsh ecosystems, where the availability of water is the cardinal parameter affecting organisms. Consequently, xerophilous microorganisms that are adapted to relatively high temperatures and radiation levels are likely to be the dominant populations in these ecosystems, including desiccation and radiation resistant phyla, such as *Actinobacteria, Proteobacteria*, and *Bacteroidetes* (Vikram et al., 2016).

**Figure 1:**
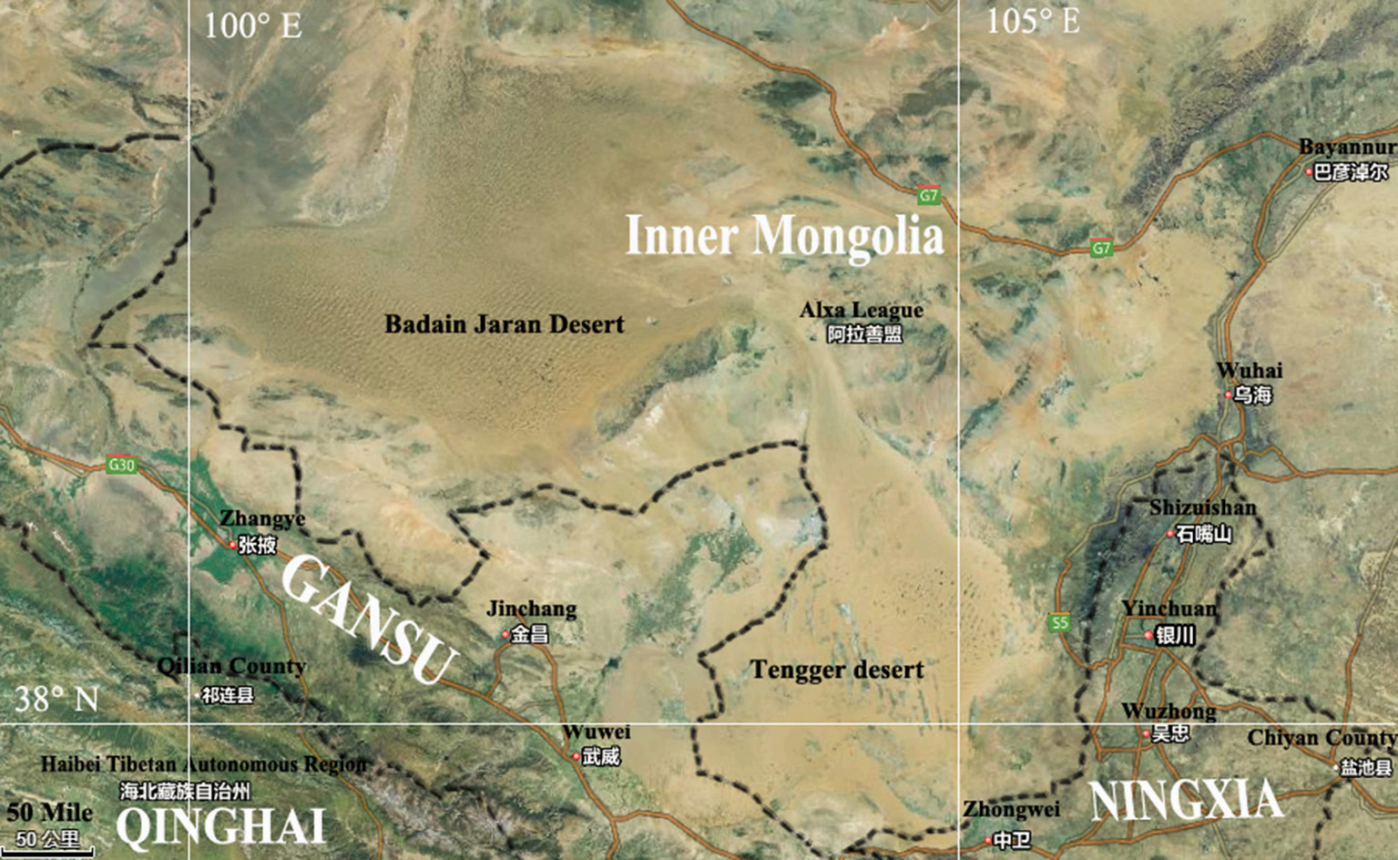
Map showing Badain Jaran desert location and Tengger desert location.

The Badain Jaran Desert (BJD) is located in the northwest region of the Alxa Plateau of the Inner Mongolia Autonomous Region (39°20′–41°30′ N, 100°–104° E), and comprises an area of 49,000 km^2^, making it the third largest desert in China (Yan et al., 2001). This region is known for its extreme continental climate and experiences the East Asian monsoon in its southwestern quadrant. The East Asian monsoon provides most of the precipitation for this area, accounting for 70% of the annualrainfall, which occurs within 10–35 days between July and September. The average annual rainfall is 150 mm while evaporation can reach as much as 1,500 mm. The Tengger Desert (TGD) is located in the southwest region of the Alashan Left Banner in the Inner Mongolia Autonomous Region and along the central border of Gansu Province (34°30′–39°00′ N, 103°–106°E). The TGD comprises a total area of about 43,000 km^2^, and is the fourth largest desert in China. A typical continental climate dominates this region, with an annual average temperature of 7–9°C, and annual rainfall of 116–148 mm that primarily occurs in July and August. The annual evaporation of this region is 3,000–3,600 mm, and the average annual wind speed is 3–4 m/s. With increasing grassland degeneration and increases in desertification, the two deserts are almost contiguous with each other in some places (Figure 1).

*Actinobacteria* are a group of bacteria with high G+C content in the genomic DNA, and well-known for producing abundant secondary active metabolites. Since Waksman discovered streptomycin from *Streptomyces* sp. in 1943, actinobacterial members have been considered as an important source of new antibiotic-producing bacteria. Currently, about 70% antibiotics in clinical use are produced by various actinomyces (Doull and Vining, 1990; Jose and Jebakumar, 2013). However, decades later, people found it is increasingly difficult to obtain novel compounds from the actinobacterial strains from normal environments. Therefore, more and more researchers proposed to find potential functional strains from various extreme environments, in which the external stress factors endow the microorganisms the unique defense mechanisms and metabolic systems, which may be more likely to produce novel antibiotics. Consequently, detecting various actinobacteria from extreme environments has become an important strategy for the discovery of new antibiotics (Phoebe et al., 2001; Wilson and Brimble, 2009). Till now, no systematic studies on actinobacteria regarding their potential abilities to produce bio-active substances and their ecological function in deserts have been reported.

The primary goal of the present research was to obtain a systematic understanding of the bacterial community structure and diversity of actinobacteria inhabiting the two immense BJD and TGD deserts of China. Further, we attempted to better characterize the distribution characteristics of actinobacterial population in these environments. Based on the above findings, we intended to optimize the isolation and cultivation strategies to explore the specific actinobacterial strains and to assess their bio-characteristics, potential functions and applications in depth.

## Materials and methods

### Sample collection

A total of 79 samples were collected from the BJD (38°20′–41°30′N, 100°–104°E). Sporadic vascular vegetation and small salt lakes characterize this desert. We thus assigned samples to the following three types based on vegetation cover and salt lake presence: sands with sporadic plants (SP), sands without any vegetation (NV), and sands around salt lakes (SL). Detailed information of samples including types, and the specific collection locations are provided in Table S1. Fifty samples were collected from the TGD, where the microbial crusts are generally categorized into the following three types: Cyanobacteria-dominated crusts (CC), moss-dominated crusts (MC), and lichen-dominated crusts (LC). Samples from the TGD also included bare sands (BS) (Sun et al., 2015), and sample information is provided in Table S2.

All samples were placed in sterilized envelopes following collection and then taken to the laboratory within 3 days of collection. The samples were immediately processed for cultivation assays and total DNA extraction after arriving at the laboratory.

### Isolation and cultivation media

#### Isolation media

The following seven media were prepared to isolate microbial strains:

M1: 1/5 strength R2A (Difco).

M2: 5 g/L yeast extract, 2 g/L cellobiose, 2 g/L CaCO_3_, 0.5 g/L MgSO4•7H2O, 1 g/L K_2_HPO_4_, and 15 g/L agar.

M3: 0.1 g/L NH _4_NO_3_, 2 g/L sodium propionate, 0.05 g/L MgSO_4_·7H_2_O, 0.1 g/L KCl, and 15 g/L agar.

M4: 1 g/L humic acid, 1 g/L asparagine, 0.01 g/L FeSO_4_, 0.5 g/L Na_2_HPO_4_, 1.7 g/L KCl, 0.02 g/L CaCO_3_, and 15 g/L agar.

M5: 2 g/L trehalose, 5 g/L yeast extract, 2 g/L CaCO_3_, 0.5 g/L MgSO_4_·7H_2_O, 1 g/L K_2_HPO_4_, and 15 g/L agar.

M6: 0.5 g/L K_2_HPO_4_, 0.25 g/L yeast extract, and 15 g/L agar.

M7: 5 g/L yeast extract, 3 g/L peptone, 10 g/L glycerol, 1.25 g/L sodium pyruvate, 1.25 g/L glycine betaine, and 15 g/L agar.

Media were adjusted to pH 7.2–7.5 using 1M HCl and/or 1M NaOH. In addition, betaine (0.125% w/v), sodium pyruvate (0.125% w/v), compound trace salts solution (0.1% v/v), and compound vitamins (0.1% w/v) were added to the media as described in Sun et al. (2015). Nystatin (25 mg/L) and potassium dichromate (50 mg/L) were added to the media to inhibit the growth of fungi and Gram negative bacteria.

#### Cultivation media

Based the obsevationof the colony diversity growth in the isolation media, the PYG medium was selected to cultivate isolates after initial isolation and consisted of 3 g/L peptone, 10 g/L glycerol, 5 g/L yeast extract, 1.25 g/L sodium pyruvate, 1.25 g/L glycine betaine, and 15 g/L agar, adjusted to pH 7.5. PYG medium was supplemented with compound trace salts solution (0.1% v/v), and compound vitamins (0.1% w/v).

#### Total DNA preparation from sand samples and PCR amplification

The 79 sand samples from BJD were pooled into seven composite samples, and the 50 sand samples from TGD were pooled into eight composite samples, according to the environments where the sands were collected. Detailed information of the 15 samples is provided in Table S3. Total genomic DNA from each of the 15 pooled samples was extracted with a PowerSoil DNA isolation kit (MoBio, USA) according to the manufacturer's protocols. Total DNA was then used as template for PCR amplification of 16S rRNA genes. The V3 to V4 hypervariable regions of 16S rRNA genes were PCR amplified using the universal bacterial primers 5′-ACTCCTACGGGAGGCAGCAG-3′ (338F) and 5′-GGACTACHVGGGTWTCTAAT-3′ (806R). PCR amplifications were performed using high fidelity TransStart Fastpfu DNA Polymerase (Transgen, China) in 20 μL reaction mixtures containing 4 μL of 5× FastPfu Buffer, 2 μL of 2.5 mM dNTPs, 0.8 μL of each primer (5 μM), 0.4 μL of FastPfu Polymerase, and 10 ng of template DNA. The reaction cycling conditions consisted of the following steps: 5 min of an initial denaturation at 94°C followed by 35 cycles of denaturation at 94°C for 30 s, 45 s of primer annealing at 55°C, 40 s of elongation at 72°C, and then a final 10 min elongation at 72°C.

#### Illumina MiSeq sequencing and raw data preprocessing

An AxyPrep DNA Gel Extraction Kit (Axygen, USA) and QuantiFluor^TM^-ST system (Promega, USA) were used to purify and quantify amplicons, respectively. Purified amplicons were pooled in equimolar concentrations and sequenced on an Illumina MiSeq platform with paired-end sequencing (2 × 250 bp). The QIIME software package (version 1.18; White et al., 2009; Caporaso et al., 2010) was used to demultiplex and quality-filter raw reads by removing reads meeting the following criteria: (a) 300 bp reads exhibiting any 50-bp sliding window with an average quality score < 20, and discarding truncated reads that were shorter than 50 bp; (b) any barcode mismatch, greater than two nucleotide mismatches in primers, and reads containing ambiguous characters (c). Lastly, paired sequences were assembled based on their overlapping sequences that were longer than 10 bp. Unassembled reads were discarded.

#### Statistical analyses

Chimeras were identified and removed using the UCHIME program (Robert et al., 2001). The Unite database (Release 7.0, http://unite.ut.ee/index.php; Kõljalg et al., 2013) was used as the taxonomic reference database to assess the taxonomic affiliation of each 16S rRNA gene sequence using a confidence threshold of 70% and the RDP Classifier (Wang et al., 2007). Sequences were clustered into operational taxonomic units (OTUs) using a 97% similarity threshold in the UPARSE program (version 7.1, http://drive5.com/uparse/). Alpha diversity and beta diversity metrics were calculated based on OTU abundances within and among samples. Specifically, alpha diversity was measured using the Chao1 richness estimator, and Shannon's diversity index, while measuring the coverage of richness using Good's coverage. Principal co-ordinates analysis (PCoA) was used to assess differences in OTU composition among samples. An analysis of similarities (ANOSIM) test was conducted to determine if sample types contained significantly different bacterial communities. Metastats and LEfSe (LDA effect size) analysis were employed to identify the bacterial groups that were significantly differentiated among sample types. Lastly, co-occurrence networks were constructed to visualize the OTU-based similarities among different communities (White et al., 2009; Caporaso et al., 2010).

#### Strains isolation, purification, maintenance, and identification

The dilution plating method was used to isolate microbial strains from the sand samples. Approximately 0.3 mL of a 10^−4^ dilution of a sand sample suspension was spread on each isolation plate. Every kind of media was parallelly spread with the suspension on two same plates and then incubated at 28°C and 45°C for 21 days, respectively. Single colonies were transferred to freshly prepared PYG plates that were supplemented with compound trace salts solution and compound vitamins, followed by subsequent purification of isolates. Purified isolates were maintained on PYG slants at 4°C and also in glycerol suspensions (20%, v/v) at −80°C (Yue et al., 2006). Genomic DNA was extracted from pure cultures and PCR amplification of 16S rRNA genes was conducted as described in Xu et al. (2003). The sequences of the isolates were compared with available 16S rRNA gene sequences from GenBank using the BLAST program and the EzBioCloud (https://www.ezbiocloud.net/) (Yoon et al., 2017).

#### Antimicrobial activity screening

Antimicrobial activities of the isolates were investigated by using media containing *Escherichia coli* ATCC 25922, *Pseudomonas aeruginosa* ATCC 27853, *Enterococcus faecalis* ATCC 29212, *Klebsiella pneumonia* subsp. *pneumoniae* ATCC 700603, and *Candida albicans* ATCC 10231, with a concentration of 10^8^ colony forming units (CFU) per mL. These assays were performed using Kirby Bauer Method with a culture broth concentration of 1% (v/v).

## Results

### Sequences and data information

The SRA accession number in DDBJ/EMBL/GenBank for the sequences data is SRP134260.

### Bacterial richness and diversity

A total of 349,374 reads with an average of 23,291 reads per sample remained after quality filtering, and were clustered into 4,298 OTUs at the 97% sequence similarity level. The alpha diversity of each sample estimated by the Chao1 estimator, and Shannon's index, in addition to the coverage estimated by Good's coverage, are provided in Table 1.

**Table 1:** Summary of high-throughput sequencing data from the 15 samples.

**Sample ID**	**Sample type**	**Number of OTUs**	**Good's coverage wstimator (%)**	**Chao1**	**Shannon**
BD201610S1	NV	799	98.65	1,046	4.95
BD201610S2	NV	938	98.51	1,145	5.3
BD201610S3	NV	925	98.08	1,190	5.22
BD201610S4	SL	804	99.31	1,051	5
BD201610S5	SL	798	99.19	1,172	4.74
BD201610S6	SP	1,129	99.49	1,294	5.6
BD201610S7	SP	1,067	99.29	1,283	4.91
SPT8001BS	BS	1,155	98.72	1,401	5.42
SPT8002BS	BS	1,165	98.72	1,405	5.67
SPT8003LC	LC	719	98.66	998	4.95
SPT8004LC	LC	824	98.89	1,015	4.34
SPT8005CC	CC	1,039	98.58	1,463	5.57
SPT8006CC	CC	653	99.34	852	4.32
SPT8007MC	MC	892	98.57	1,108	5.34
SPT8008MC	MC	756	98.98	1,063	3.42

*BS, Bare sand; CC, Cyanobacteria-dominated crusts; MC, Moss-dominated crusts; LC, Lichen-dominated crusts; SP, sand soil with sporadic plants; NV, sands without any vegetation; SL, sand sample from around salt lakes*.

**Figure 2:**
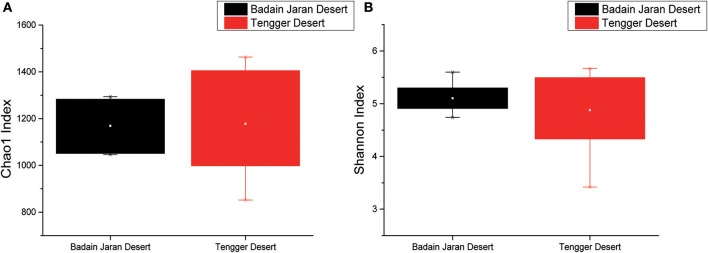
Statistical comparisons of Chao1 and Shannon indices between two desert sand samples. **(A)** Values of Chao1 index; **(B)** values of Shannon index.

Rarefaction analyses using the Shannon index as a diversity metric indicated that our sequencing efforts covered nearly all of the diversity that would be expected to be found in these samples (Figure S1). On average, 923 (95% CI: 799–1,029) and 900 (95% CI: 653–1,165) OTUs were found in each of the BJD and TGD samples, respectively. Samples from the desert sands exhibited almost identical Chao1 and Shannon index values, as indicated by ANOVA tests, suggesting no significant difference in the richness or diversity of bacteria between the two deserts (Figure 2).

**Figure 3:**
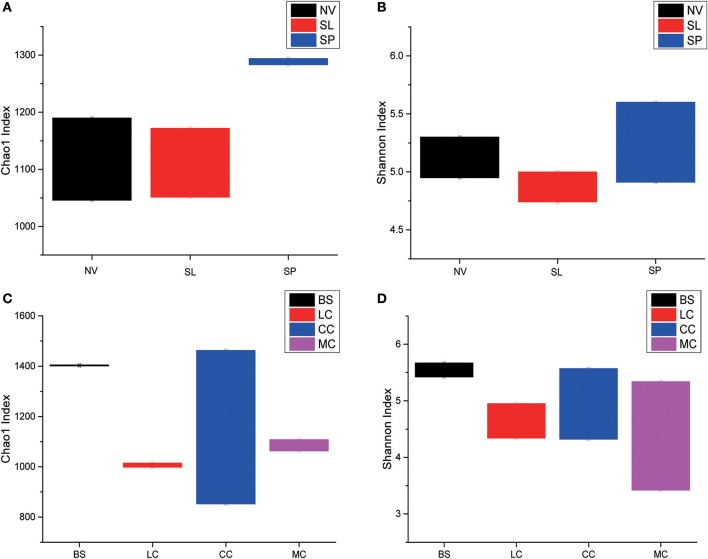
Statistical comparisons of Chao1 and Shannon indices among three types of Badain Jaran desert sand samples (NV, SP, and SL) and four types of Tengger desert sand samples (BS, LC, MC, and CC). **(A)** Values of Chao1 in of BJD; **(B)** values of Shannon index of BJD; **(C)** values of Chao1 of TGD; **(D)** values of Shannon index of TGD.

Among the BJD samples, SP samples had the highest Chao1 values, whereas samples from NV, SL and SP did not significantly differ. Among the TGD samples, bacterial richness and diversity was slightly higher in BS samples compared to the others, which was reflected in higher Chao1 and Shannon index values (Figure 3).

### Bacterial and actinobacterial community structure

**Figure 4:**
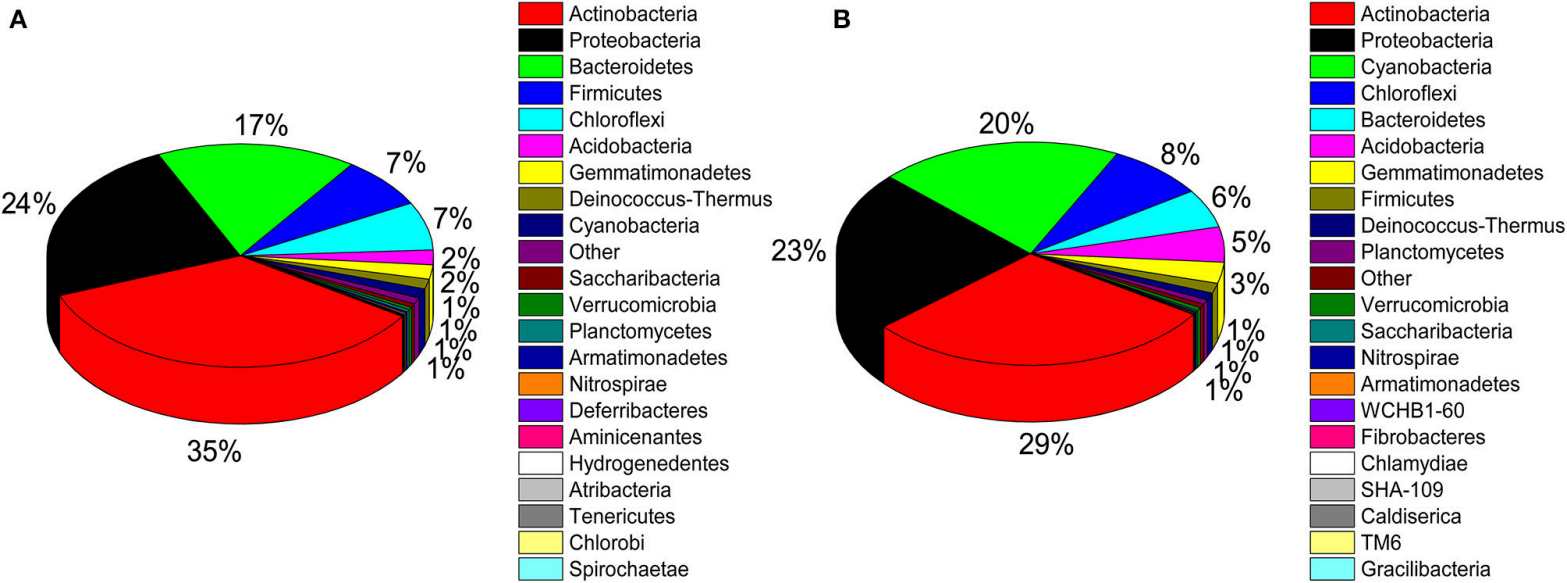
Pie chart showing the percentage of relative abundance of bacterial communities from two desert sand samples in HTS method. **(A)** Badain Jaran desert sands; **(B)** Tengger Desert sands.

**Figure 5:**
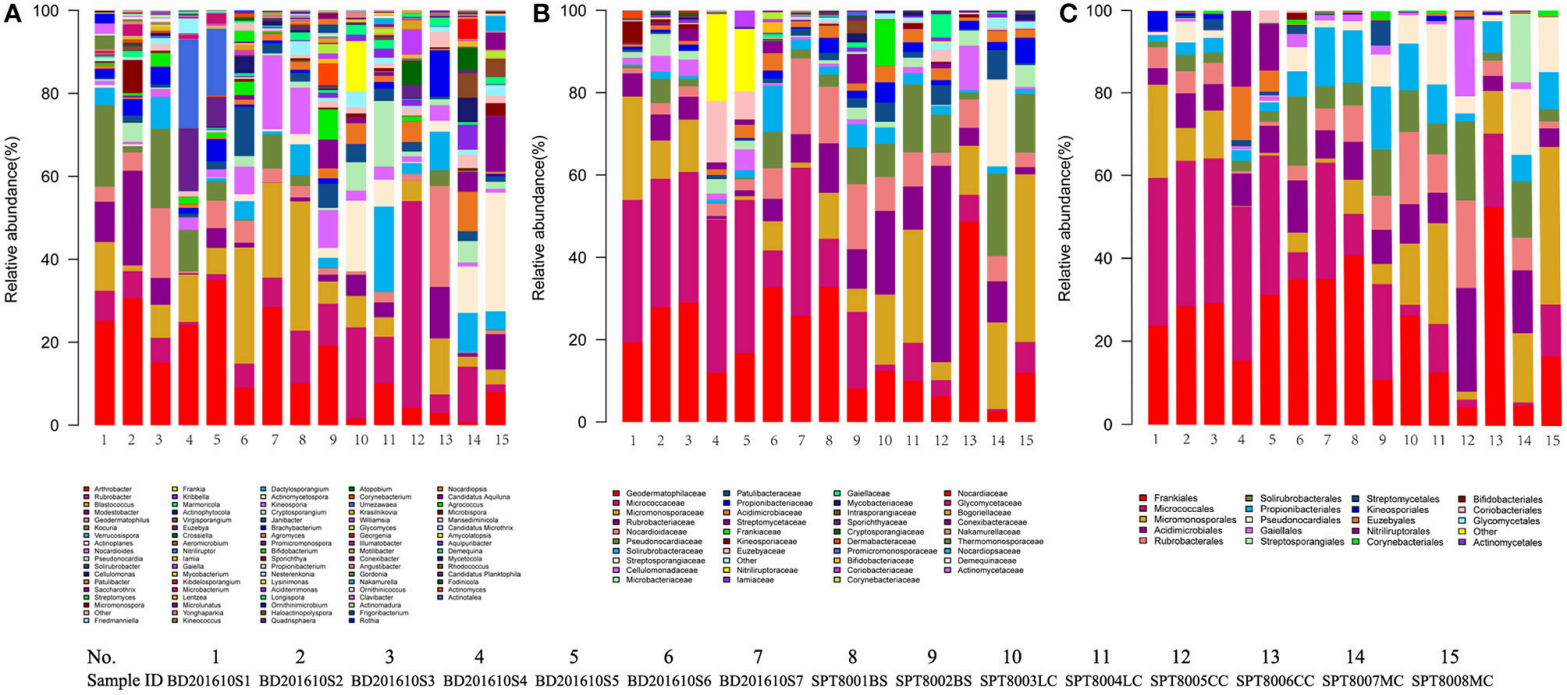
The relative abundances of different actinobacterial taxon level of two desert sand samples in HTS method. **(A)** Genus taxon level; **(B)** family taxon level; **(C)** order taxon level.

A total of 32 phyla, 97 orders, and 470 genera were identified in BJD samples, while 28 phyla, 73 orders, and 302 genera were identified in TGD samples. *Actinobacteria* dominated the bacterial communities of both deserts (35 and 29% relative abundance in BJD and TGD, respectively), followed by *Proteobacteria, Bacteroidetes*, and *Chloroflexi* (Figure 4). *Frankiales, Micrococcales, Micromonosporales*, and *Acidimicrobiales* were the most abundant orders within the 15 sand samples. Among these, the families *Geodermatophilaceae, Micrococcaceae, Micromonosporaceae*, and *Rubrobacteriaceae* were the predominant actinobacterial populations in both deserts. Lastly, the most abundant genera were *Arthrobacter* and *Rubrobacter* in the actinobacterial community, followed by *Blastococcus, Modestobacter*, and *Geodermatophilus*, which are members of the *Geodermatophilaceae* (Figure 5). In BJD, *Micrococcaceae* was much more prevalent than *Geodermatophilaceae*, while the reverse was observed in TGD samples. *Arthrobacter* was the most abundant genus in BJD samples and the second most abundant genus in TGD samples. *Kocuria* was a dominant genus in BJD samples, but only a few *Kocuria* OTUs were detected in TGD samples. Conversely, *Actinoplanes* were more abundant in TGD samples than in BJD samples. In addition to these dominant genera, a large number of rare bacterial genera were also detected (we defined rare bacterial groups as less than 0.1% abundance of communities), and included the genera *Conexibacter, Longispora, Dactylosporangium, Umezawaea, Demequina, Janibacter*, and *Motilibacter* in BJD and genera *Ornithinimicrobium, Angustibacter, Nakamurella, Aquiluna, Williamsia, Amycolatopsis*, and *Kineosporia* in TGD (Figure 5).

**Figure 6:**
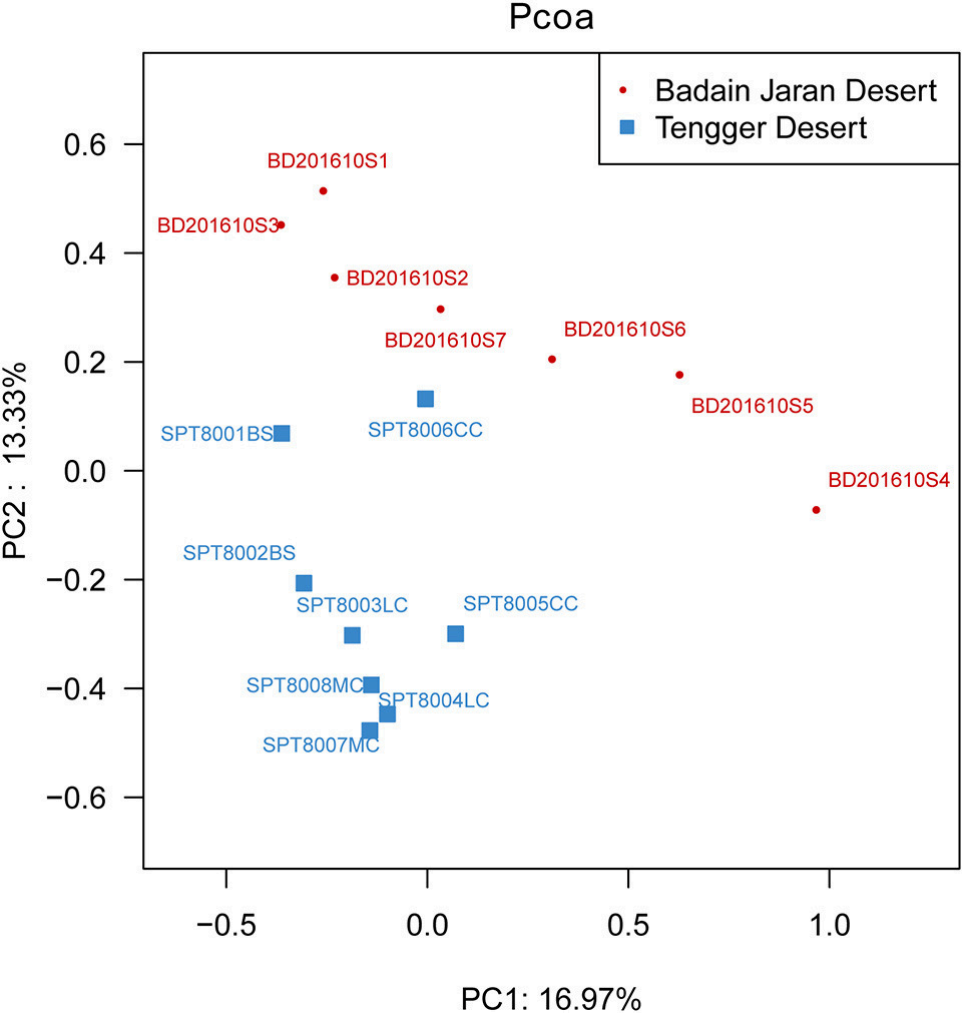
PCoA diagram showing the significant difference in different desert samples.

Metastats analysis indicated that the composition of actinobacterial genera in the two deserts were not significantly different (*P* = 1) (Table S4). However, different sand types represented distinct ecosystems, and generally, bacterial community composition was more strongly correlated with sampling site rather than micro-ecosystem type (Figure 6).

**Figure 7:**
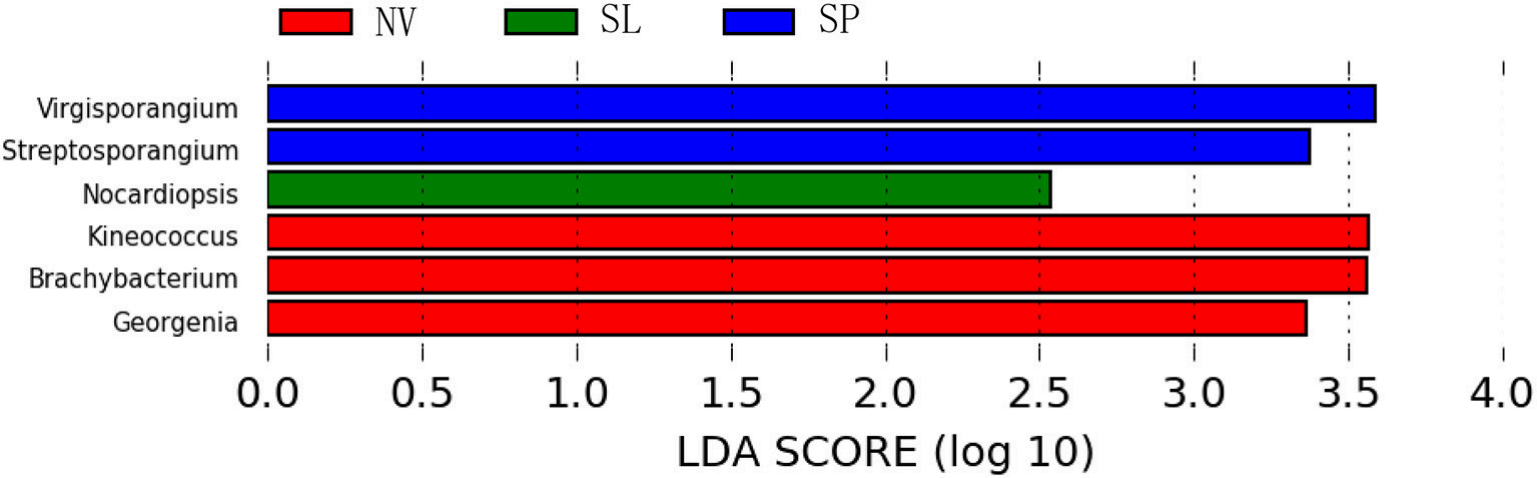
The LEfSe analysis indicating the significant difference among three types of Badain Jaran desert sand samples.

In particular, ANOSIM (*R* = 0.328, *p* = 0.001) indicated that community structures were not different between samples taken from sampling sites that were close together. LEfSe analyses indicated that there were some significant differences in community composition among the three sample types from BJD. For example, the abundances of the genera *Virgisorangium, Nocardiopsis, Brachybacterium, Kineococcus, Georgenia*, and *Streptosporangium* were highly differential among sample types within BJD (Figure 7). However, no significant differences in the abundances of these genera were observed for samples from the TGD. The *Geodermatophilaceae* and *Micrococcaceae* were the dominant families in NV, SL, SP, and BS samples, whereas *Micromonosporaceae* were more abundant in the LC, CC, and MC samples. Further, *Pseudonocardiaceae* were more abundant in NV, SP, BS, LC, CC, and MC samples, but not in SL samples. *Arthrobacter* was the predominant genus in NV, SL, SP, and BS samples, but not in LC, CC, or MC samples. Lastly *Rubrobacter* were abundant in LC, CC and MC samples, but not NV, SL, SP, or BS samples (Figure S2).

**Figure 8:**
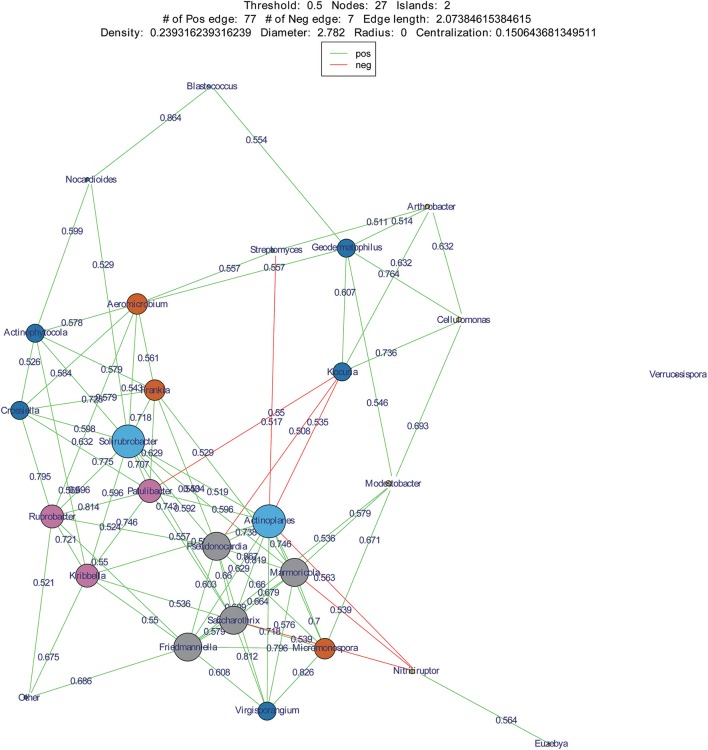
Co-occurrence networks of dominant genera in two deserts. Each node means a dominant genus, each edge means two correlated genera with an absolute Spearman's correlation above 0.5 at a 0.05 significance level; the edge length is based on Bray-curtis distance and the node size means the number of its connection with other genera.

We used co-occurrence network analysis to identify correlations between different genera among samples. A total of 27 nodes and 84 correlation edges were used to represent the dominant genera (>1% relative abundance). Correlation analysis indicated 77 positive and seven negative correlations. The genera *Solirubrobacter* and *Actinoplanes* exhibited the most correlations (12 edges for each), followed by the *Pseudonocardia, Marmoricola, Saccharothrix*, and *Friedmanniella* which each comprised 10 edges. These results indicated that the six genera were likely to be the key actinobacterial members in the sand communities of the two deserts. Among the family *Geodermatophilaceae*, three genera appeared to also be important with *Geodermatophilus* exhibiting six edges, *Modestobacter* with five, and *Blastococcus* with two (Figure 8).

### Isolation of actinobacterial strains

**Figure 9:**
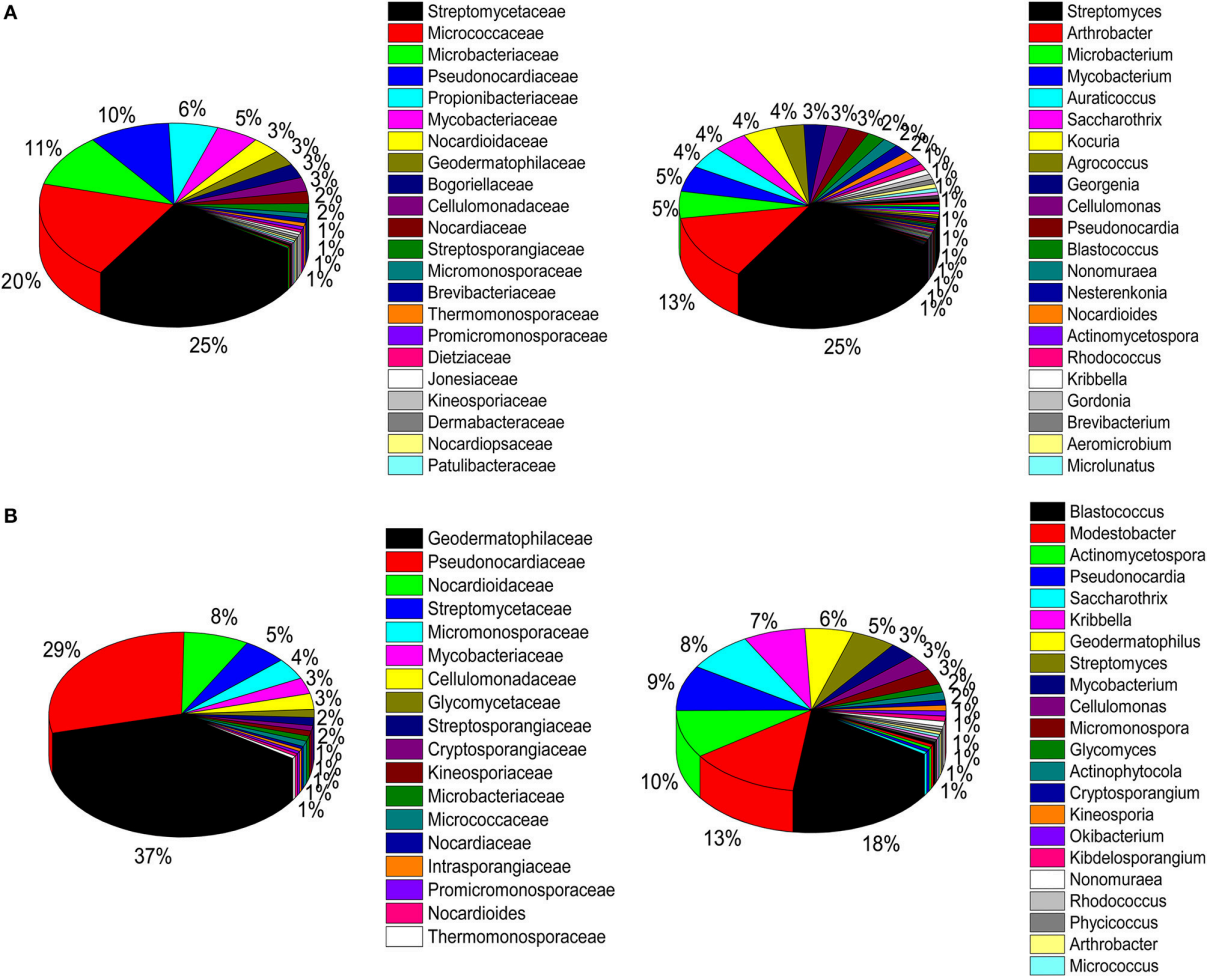
Pie chart showing the percentage of relative abundance of bacterial communities from two desert sand samples in CULD method. **(A)** Badain Jaran desert sands; **(B)** Tengger desert sands. The sequences of the bacteria were assigned to family (1/3) and genus level (2/4).

A total of 786 actinobacterial strains were isolated and identified from the BJD samples. 16S rRNA gene sequencing indicated that these 786 strains belonged to 30 families and 73 genera (Figure S3A). In addition, 376 actinobacterial strains were isolated from TGD samples, comprising 18 families and 29 genera (Figure S3B). 16S rRNA gene sequencing indicated that members of *Geodermatophilaceae* were the major cultivated actinobacterial taxa in both of these deserts, which is consistent with the cultivation-independent analyses. Moreover, the taxonomic distributions of isolates were highly similar to those of the cultivation-independent analyses. *Arthrobacter* spp. and *Kocuria* spp. were the primary cultivated genera, and comprised 13.4 and 4.1% of the actinobacterial community isolates in BJD, respectively (Figure S4; Figure 9). *Streptomyces* spp. were also dominant among the isolated genera, comprising 25% of BJD isolates and 17.5% of TGD isolates. In addition, some low abundance taxa that were detected using cultivation-independent analysis including *Deinococcus* spp., *Skermanella* spp., *Actinophytocola* spp., *Patulibacter* spp., *Rufibacter* spp., and *Geminicoccus* spp. were also isolated.

### Comparative analysis of HTS and CULD results

**Figure 10:**
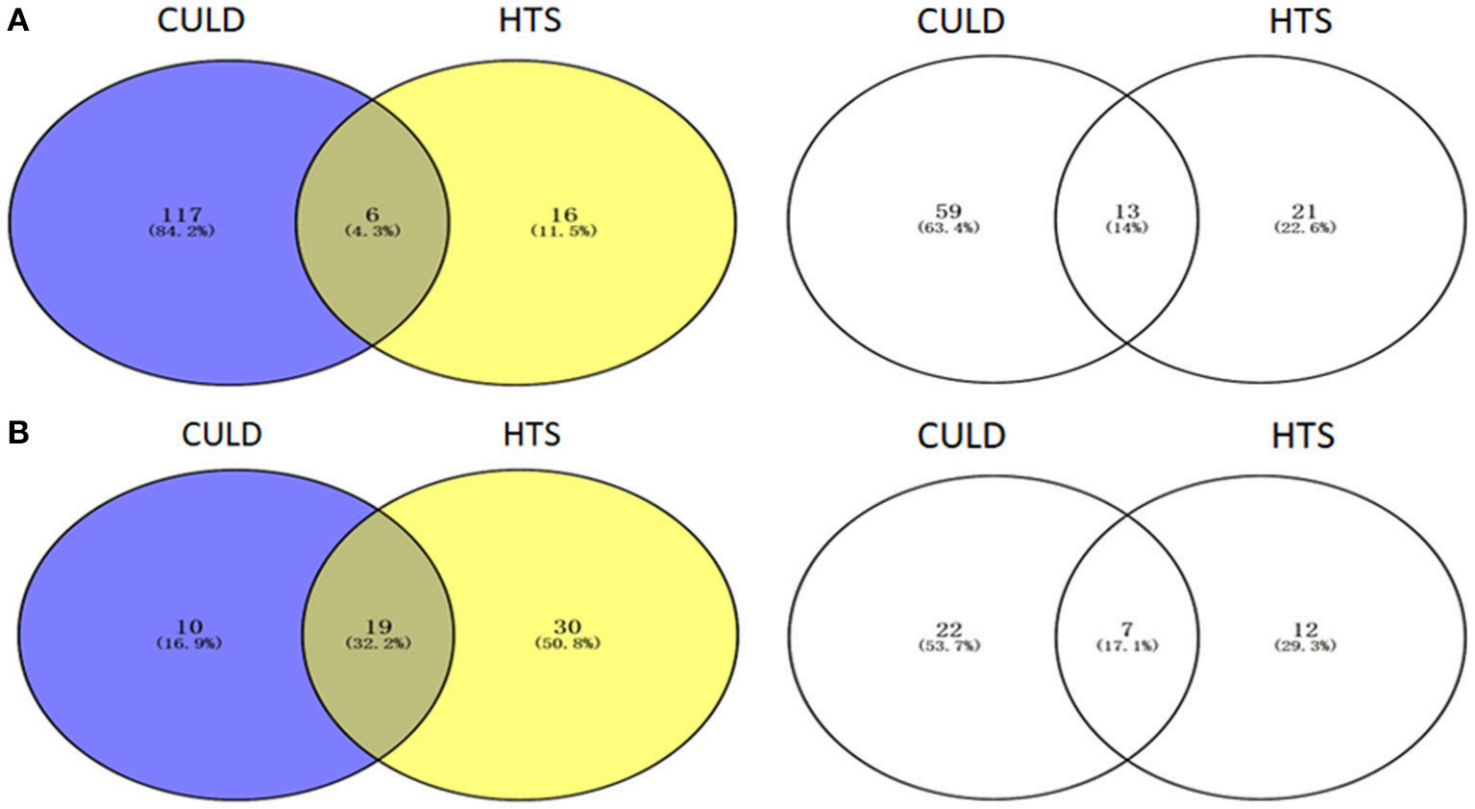
Venn diagrams illustrating the number of genera between High-throughput sequencing analysis and Cultivation-dependent method. **(A)** Badain Jaran desert sands; **(B)** Tengger desert sands. The Venn diagrams were compared by dominant genera (1/3) and rare genera (2/4).

Of the dominant genera (>1%), 4.3 and 32.2% were detected using the two methodologies in BJD and TGD samples, respectively (Figure 10). In addition, 14.0 and 17.1% of the rare genera (<0.1%) were identified by both methodologies in BJD and TGD, respectively. However, there were some genera that were only detected by either cultivation-dependent (CULD) or high-throughput sequencing (HTS) analysis (Figure 10). These results indicate that the composition of the native microbial communities are not only driven by the dominant groups, but also rare taxa that are active and integral members of community structure.

### Actinobacterial members in desert sands

**Figure 11:**
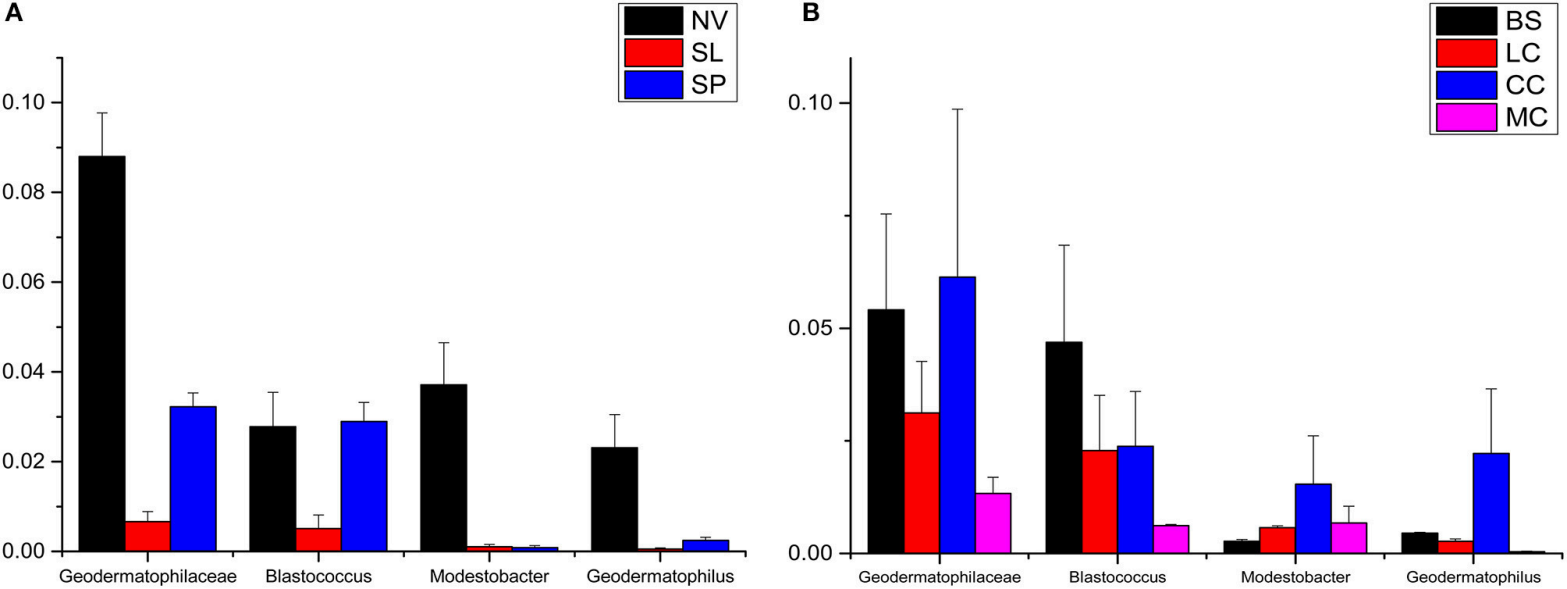
The relative abundances of Geodermatophilaceae in different types of the two deserts. **(A)** Badain Jaran Desert sand samples; **(B)** Tengger Desert sand samples.

**Figure 12:**
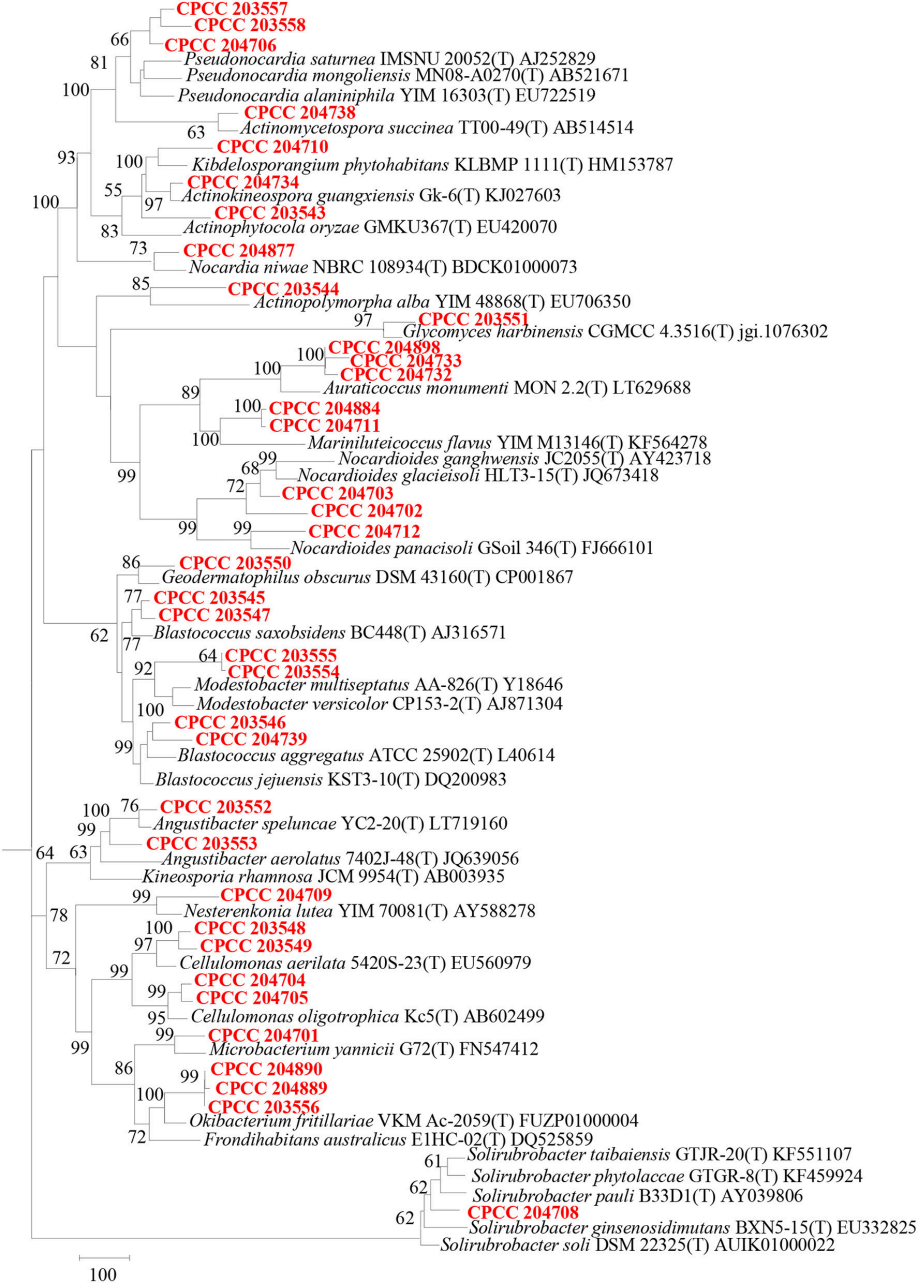
Dendrogram based on 16S rRNA gene sequences analysis of 37 novel species and some relevant strains in the two deserts. Bar, 100 substitutions per nucleotide position.

*Geodermatophilaceae* populations were the dominant group in both the BJD and TGD deserts, comprising 27.4 and 18.2% of the *Actinobacteria* according to cultivation-independent analyses. The genera *Blastococcus, Modestobacter*, and *Geodermatophilus* exhibited nearly equivalent diversity in the two deserts (Figure S4). However, *Blastococcus* was more ubiquitous and abundant in all samples, compared to the other two genera. *Modestobacter* was more abundant in NV, MC, and CC samples, while OTUs associated with this genus were rare in SL, SP, and BS samples. *Geodermatophilus* was abundant in NV, SP, and CC samples, but not in LC or MC samples. Generally, NV, SP, BS, and CC samples harbored the most abundant *Geodermatophilaceae* communities (Figure 11). Futher, more *Geodermatophilaceae* isolates were obtained form the NV and BS samples, which could be regarded as more oligotrophic environments. In total, 52 *Geodermatophilaceae* strains were obtained from BJD, including 36 *Blastococcus* spp., six *Geodermatophilus* spp. and 10 *Modestobacter* spp. In addition, 34 *Blastococcus* spp., 11 *Geodermatophilus* spp., and 25 *Modestobacter* spp. were obtained from TGD. Importantly, 21 actinobacterial isolates from BJD representing novel species within the following 15 genera were obtained: *Actinokineospora, Actinomycetospora, Auraticoccus, Blastococcus, Cellulomonas, Kibdelosporangium, Mariniluteicoccus, Microbacterium, Nesterenkonia, Nocardia, Nocardioides, Pseudonocardia*, and *Solirubrobacter*. Likewise, 16 novel taxa were isolated from TGD that were distributed among 11 genera (Figure 12). These novel actinobacterial taxa are worthy of further bioprospecting studies.

### Antimicrobial activities

Five-hundred actinobacterial isolates from BJD were screened for antimicrobial activities. Of these, 1.6% of the isolates exhibited antimicrobial activity against *Escherichia coli* ATCC 25922, 3.5% against *Enterococcus faecalis* ATCC 29212, 1.7% against *Klebsiella pneumonia* subsp. *pneumoniae* ATCC 700603, 1.2% against *Pseudomonas aeruginosa* ATCC 27853, and 2.2% against *Candida albicans* ATCC 10231. Overall, 10.36% of the tested isolates from BJD exhibited antimicrobial activity in one or more screens.

## Discussion

With the rapid technological innovation in molecular biology in recent years, various new techniques, and in particular high-throughput cultivation-independent approaches, have been extensively applied in microbial ecology research. These new methodologies have greatly expanded our knowledge of bacterial community composition. Consequently, numerous studies have shown that microbial diversity is far higher and more complex than previously thought. 16S rRNA gene cloning and denaturing gradient gel electrophoresis (DGGE) have been traditionally used to identify uncultured microbial community structures in the past. However, these methods are particularly susceptible to 16S rRNA gene copy numbers, and the methods are both complicated and costly. High-throughput sequencing precludes the need to build clone libraries, and the efficiency associated with HTS sequencing approaches and the widespread adoption of the Illumina Miseq sequencing platform has accelerated analytical capabilities. Consequently, HTS analysis on the Illumina Miseq platform has provided comprehensive and efficient analyses of environmental microbial communities. (Navarro-Noya et al., 2013; Poulsen et al., 2013; Sul et al., 2013; Yu et al., 2013; Wu et al., 2014). Claesson et al. (2010) conducted a comprehensive analysis of 9 variable regions of the 16S rRNA gene. The results showed that V1 and V9 had the worst effects, followed by V7 and V8 regions, while V3, V4, and V5 regions were relatively better. In addition, by comparing the regions of V1/V2, V2/V3, V3/V4, V5/V6, and V7/V8 in series, the accuracy of the tandem region V3/V4 and V4/V5 were the highest. Considering the classification efficiency (CE), the V3 / V4 region had significant amplification preference relative to other regions (Claesson et al., 2010). Here, we analyzed the bacterial communities of two Chinese deserts using HTS, and accordingly, modified the media and temperature for isolation and cultivation the targeted actinobacterial strains. Our results provide evidence for a number of actinobacterial strains that are specific for these desert sands and were isolated and identified via cultivation-dependent and -independent analyses. Importantly, these results describe the diversity and composition of actinobacterial communities in different sand types, which has not been systematically investigated in these two deserts (Sun et al., 2015; Li et al., 2016).

Comparative results from the two deserts and the different mico-ecosystems indicated that geographical barrier contributed much more in shaping the bacterial communities than micro-ecological types. On the other side, even in a certain same terrain, the indigenous microorganisms' ecological service function resulted in discriminating micro-ecosystem.

Deserts are typical of extreme, harsh ecosystems, where the availability of water is the cardinal parameter affecting organisms. Consequently, xerophilous microorganisms that are adapted to relatively high temperatures and radiation levels are likely to be the dominant populations in these ecosystems. *Actinobacteria* are a ubiquitously distributed bacterial phylum inhabiting diverse terrestrial ecosystems. Based on this observation, we approved that *Actinobacteria* is the predominant microorganisms in desert sand microbial communities using HTS and CULD data. Generally, *Actinobacteria*, characterized by high genomic GC content, complex peptidoglycan layer containing in the thick cell wall, motility with spore-bearing cells and pigmentation in colonies, may have evolved into some genetic and metabolic functions to help them inhabit in the deserts with drought and high ultraviolet radiation (Mohammadipanah and Wink, 2012).

A total of 4,298 OTUs were obtained using cultivation-independent HTS, indicating a high level of bacterial diversity in these desert environments. Importantly, the cultivation-independent and -dependent analyses provide evidence for valuable microbial resources that warrant further research. In particular, some specific, novel *Actinobacteria* members and rare microbial groups were identified that could serve as highly valuable microbial resources. OTUs affiliated with the *Geodermatophilaceae* were frequently detected among the 15 sample types analyzed here, which is consistent with our previous supposition that *Geodermatophilaceae* are ubiquitous within arid deserts (Sun et al., 2015). *Geodermatophilaceae* have been detected from various relatively extreme environments, including stone habitats (Salazar et al., 2006; Chouaia et al., 2012; Gtari et al., 2012; Normand et al., 2012), dry hot valleys (Nie et al., 2012), and arid sand from deserts (Montero-Calasanz et al., 2012; Montero-Calasanz C. et al., 2013; Montero-Calasanz M. C. et al., 2013; Saul-Tcherkas et al., 2013), among others. Thus, *Geodermatophilaceae* strains may be pioneer microorganisms that play a key role in shaping the bacterial community structure in these harsh environments. Furthermore, to some extent, in these desert habits, the microbial community structures may always be doomed from some pioneer population's colonization, where the core groups may promote or inhabit the subsequent members' occurrence and prosper.

*Actinoplanes* and *Kocuria* were abundant members of their communities and were also two key genera of the desert sand communities based on co-occurrence network analyses. Interestingly, *Actinoplanes* and *Kocuria* were negatively correlated in the co-occurrence network. A large number of *Kocuria* spp. were isolated from our cultivation assays (21 strains) from sand samples, yet only a few *Actinoplanes* strains were obtained. In addition, *Streptomyces* was an abundant component of both the HTS and cultivation assays. However, *Streptomyces* and *Actinoplanes* were negatively correlated in the co-occurrence network analysis. These results point to a potentially intriguing negative interaction between *Streptomyces, Kocuria* and *Actinoplanes* that may explain the paucity of *Actinoplanes* isolates that were obtained. Future research should investigate the potential for supplements that inhibit the growth of *Streptomyces* and *Kocuria* in order to promote the isolation of *Actinoplanes*. Regardless, our results suggest the presence of valuable microbial strains, particularly rare community members that are present within these desert sands, and which may hold great significance in understanding the ecology of these systems or in novel microbiological drug research. The analyses here, particularly the co-occurrence networks, provide a framework from which to design isolation media that may promote the growth of these potentially valuable taxa.

## Author contributions

YS carried out the experiments and prepared the manuscript. Y-LS helped prepare some experiments. TZ, L-YY, and HW contributed in sampling from deserts. HS helped correct the writing. Y-QZ is responsible for designing the research and preparing the manuscript.

### Conflict of interest statement

The authors declare that the research was conducted in the absence of any commercial or financial relationships that could be construed as a potential conflict of interest.
