# Supplementary material for: Diversity of Bacteria and the Characteristics of Actinobacteria Community Structure in Badain Jaran Desert and Tengger Desert of China
Source: Front Microbiol. 2018 May 23;9:1068. doi: 10.3389/fmicb.2018.01068 (PMC5974926; doi:10.3389/fmicb.2018.01068)
Supplement: Supplementary file 1 [file Data_Sheet_1.docx]

**Table S1.** The sample information of Badain Jaran Desert

| **Badain Jaran Desert** | | | |
| --- | --- | --- | --- |
| **sample number** | **sample type** | **site information** | **altitude** |
| 23003 | SP | 39°50'00"N;102°00'39"E | 1224 |
| 23014 | SP | 39°31'16"N;101°44'22"E | 1382 |
| 23007 | SP | 39°39'58"N;101°53'25"E | 1367 |
| 23001 | SP | 39°54'43"N;102°04'02"E | 1240 |
| 23005 | SP | 39°45'51"N;101°57'09"E | 1320 |
| 23009 | SP | 39°34'24"N;101°47'56"E | 1286 |
| 23011 | SP | 39°33'38"N;101°47'26"E | 1299 |
| 22032 | SP | 39°48'22"N;101°58'17"E | 1272 |
| 22026 | SP | 39°36'59"N;101°51'37"E | 1350 |
| 22025 | SP | 39°31'23"N;101°44'27"E | 1388 |
| 22023 | SP | 39°31'23"N;101°44'27"E | 1388 |
| 22030 | SP | 39°40'59"N;101°54'09"E | 1291 |
| 22033 | SP | 39°48'22"N;101°58'17"E | 1272 |
| 22027 | SP | 39°36'59"N;101°51'37"E | 1350 |
| 23002 | SP | 39°54'43"N;102°04'02"E | 1240 |
| 23004 | SP | 39°50'00"N;102°00'39"E | 1224 |
| 23006 | SP | 39°45'51"N;101°57'09"E | 1320 |
| 23015 | SP | 39°31'16"N;101°44'22"E | 1382 |
| 23008 | SP | 39°39'58"N;101°53'25"E | 1367 |
| 23010 | SP | 39°34'24"N;101°47'56"E | 1286 |
| 23013 | SP | 39°33'38"N;101°47'26"E | 1299 |
| 23016 | SP | 39°31'16"N;101°44'22"E | 1382 |
| 23017 | SP | 39°31'16"N;101°44'22"E | 1382 |
| 22028 | SP | 39°40'59"N;101°54'09"E | 1291 |
| 22048 | SL | 39°51'53"N;102°02'06"E | 1147 |
| 22051 | SL | 39°51'53"N;102°02'06"E | 1147 |
| 22040 | SL | 39°50'51"N;102°01'20"E | 1140 |
| 22041 | SL | 39°50'51"N;102°01'20"E | 1140 |
| 22043 | SL | 39°50'51"N;102°01'20"E | 1140 |
| 22049 | SL | 39°51'53"N;102°02'06"E | 1147 |
| 22052 | SL | 39°51'53"N;102°02'06"E | 1147 |
| 22042 | SL | 39°50'51"N;102°01'20"E | 1140 |
| 22053 | SL | 39°51'53"N;102°02'06"E | 1147 |
| 22047 | SL | 39°51'53"N;102°02'06"E | 1147 |
| 22050 | SL | 39°51'53"N;102°02'06"E | 1147 |
| 22054 | SL | 39°55'45"N;102°05'48"E | 1235 |
| 22055 | SL | 39°55'45"N;102°05'48"E | 1235 |
| 22060 | SL | 39°56'40"N;102°05'39"E | 1143 |
| 22061 | SL | 39°56'40"N;102°05'39"E | 1143 |
| 22062 | SL | 39°56'40"N;102°05'39"E | 1143 |
| 22064 | SL | 39°56'29"N;102°05'40"E | 1149 |
| 22063 | SL | 39°56'29"N;102°05'40"E | 1149 |
| 22035 | SL | 39°48'14"N;101°59'27"E | 1150 |
| 22038 | SL | 39°48'14"N;101°59'27"E | 1150 |
| 22036 | SL | 39°48'14"N;101°59'27"E | 1150 |
| 22039 | SL | 39°48'14"N;101°59'27"E | 1150 |
| 21035 | NV | 39°24'31"N;102°23'03"E | 1425 |
| 21011 | NV | 39°21'34"N;101°3'42"E | 1510 |
| 21046 | NV | 39°27'49"N;102°51'36"E | 1275 |
| 21045 | NV | 39°24'06"N;102°37'00"E | 1285 |
| 21040 | NV | 39°24'15"N;102°31'25"E | 1324 |
| 21026 | NV | 39°18'45"N;101°55'37"E | 1580 |
| 21016 | NV | 39°17'21"N;101°19'19"E | 1450 |
| 21020 | NV | 39°12'1"N;101°28'31"E | 1420 |
| 21015 | NV | 39°17'21"N;101°19'19"E | 1450 |
| 22001 | NV | 39°51'17"N;103°35'19"E | 1460 |
| 22020 | NV | 39°24'44"N;102°42'45"E | 1254 |
| 22012 | NV | 39°37'59"N;103°04'58"E | 1246 |
| 22008 | NV | 39°44'04"N;103°15'09"E | 1263 |
| 22015 | NV | 39°31'12"N;103°00'15"E | 1241 |
| 21022 | NV | 39°12'1"N;101°28'31"E | 1420 |
| 21031 | NV | 39°21'19"N;102°19'20"E | 1550 |
| 21013 | NV | 39°21'34"N;101°3'42"E | 1510 |
| 21017 | NV | 39°17'21"N;101°19'19"E | 1450 |
| 21036 | NV | 39°24'31"N;102°23'03"E | 1425 |
| 21048 | NV | 39°27'49"N;102°51'36"E | 1275 |
| 21044 | NV | 39°24'06"N;102°37'00"E | 1285 |
| 21032 | NV | 39°21'19"N;102°19'20"E | 1550 |
| 21009 | NV | 39°15'15"N;100°53'23"E | 1690 |
| 21025 | NV | 39°18'45"N;101°55'37"E | 1580 |
| 21002 | NV | 39°10'38"N;100°35'15"E | 1690 |
| 22003 | NV | 39°51'17"N;103°35'19"E | 1460 |
| 22010 | NV | 39°44'04"N;103°15'09"E | 1263 |
| 22018 | NV | 39°31'12"N;103°00'15"E | 1241 |
| 22014 | NV | 39°37'59"N;103°04'58"E | 1246 |
| 22021 | NV | 39°24'44"N;102°42'45"E | 1254 |
| 21039 | NV | 39°24'15"N;102°31'25"E | 1324 |
| 21001 | NV | 39°10'38"N;100°35'15"E | 1690 |
| 21007 | NV | 39°15'15"N;100°53'23"E | 1690 |

**Table S2.** The sample information of Tengger Desert

| **Tengger Desert** | | | |
| --- | --- | --- | --- |
| **sample number** | **sample type** | **site information** | **altitude** |
| BCL12001 | BS | 37°25'38.72"N;104°35'8.13"E | 1701 |
| MSY12018 | MS | 37°27'38.50"N;104°59'58.79"E | 1329 |
| BSY12035 | BS | 37°27'38.66"N;104°59'58.52"E | 1329 |
| MCL12002 | MS | 37°25'37.85"N;104°35'8.26"E | 1701 |
| MSY12019 | MS | 37°27'38.36"N;104°59'59.10"E | 1329 |
| CSY12036 | CC | 37°27'38.12"N;104°59'58.79"E | 1329 |
| BCL12003 | BS | 37°25'38.89"N;104°35'9.01"E | 1701 |
| MSY12020 | MS | 37°27'38.18"N;104°59'59.54"E | 1329 |
| CSY12037 | CC | 37°27'37.81"N;104°59'59.47"E | 1329 |
| CCL12004 | CC | 37°25'38.65"N;104°35'7.67"E | 1701 |
| MSY12021 | MS | 37°27'38.04"N;104°59'59.84"E | 1329 |
| CSY12038 | CC | 37°27'37.76"N;104°59'59.72"E | 1329 |
| MCL12005 | MS | 37°25'39.09"N;104°35'7.97"E | 1701 |
| MSY12022 | MS | 37°27'37.86"N;105°00'0.17"E | 1329 |
| CSY12039 | CC | 37°27'37.60"N;104°59'59.72"E | 1329 |
| CYW12006 | CC | 37°25'30.86"N;104°43'52.00"E | 1698 |
| MSY12023 | MS | 37°27'37.89"N;105°00'0.51"E | 1329 |
| CSY12040 | CC | 37°27'37.40"N;104°59'59.86"E | 1329 |
| LYW12007 | LC | 37°25'30.76"N;104°43'53.52"E | 1698 |
| MSY12024 | MS | 37°27'38.09"N;105°00'0.28"E | 1329 |
| CSY12041 | CC | 37°27'37.45"N;105°00'0.13"E | 1329 |
| LYW12008 | LC | 37°25'29.83"N;104°43'53.65"E | 1698 |
| MSY12025 | MS | 37°27'38.29"N;105°00'0.04"E | 1329 |
| BSY12042 | BS | 37°27'37.29"N;104°59'59.86"E | 1329 |
| BYW12009 | BS | 37°25'30.14"N;104°43'51.19"E | 1698 |
| BSY12026 | BS | 37°27'38.63"N;104°59'59.68"E | 1329 |
| BSY12043 | BS | 37°27'37.32"N;104°59'59.59"E | 1329 |
| MYW12010 | MS | 37°25'31.17"N;104°43'51.13"E | 1698 |
| CSY12027 | CC | 37°27'38.87"N;104°59'59.44"E | 1329 |
| CSY12044 | CC | 37°27'37.34"N;104°59'59.18"E | 1329 |
| BYW12011 | BS | 37°25'31.35"N;104°43'52.09"E | 1698 |
| MSY12028 | MS | 37°27'39.09"N;104°59'59.24"E | 1329 |
| BSY12045 | BS | 37°27'37.36"N;104°59'58.79"E | 1329 |
| BHW12012 | BS | 37°27'3.06"N;104°47'41.19"E | 1619 |
| MSY12029 | MS | 37°27'39.16"N;104°59'59.63"E | 1329 |
| CSY12046 | CC | 37°27'37.55"N;104°59'58.97"E | 1329 |
| BHW12013 | BS | 37°27'3.45"N;104°47'42.21"E | 1619 |
| CSY12030 | CC | 37°27'39.03"N;104°59'59.89"E | 1329 |
| CSY12047 | CC | 37°27'37.76"N;104°59'58.65"E | 1329 |
| MHW12014 | MS | 37°27'3.67"N;104°47'40.92"E | 1619 |
| MSY12031 | MS | 37°27'38.83"N;105°00'0.15"E | 1329 |
| BSY12048 | BS | 37°27'37.96"N;104°59'58.32"E | 1329 |
| LHW12015 | LC | 37°27'4.05"N;104°47'41.63"E | 1619 |
| BSY12032 | BS | 37°27'38.54"N;105°00'0.61"E | 1329 |
| BSY12049 | BS | 37°27'37.90"N;104°59'58.03"E | 1329 |
| LHW12016 | LC | 37°27'3.24"N;104°47'41.36"E | 1619 |
| MSY12033 | MS | 37°27'38.38"N;105°00'0.81"E | 1329 |
| CSY12050 | CC | 37°27'37.99"N;104°59'59.11"E | 1329 |
| MSY12017 | MS | 37°27'38.52"N;104°59'59.89"E | 1329 |
| BSY12034 | BS | 37°27'38.17"N;105°00'0.06"E | 1329 |

**Table S3**. Detail information of the 15 samples

| sample number | sample type | sample resource |
| --- | --- | --- |
| SPT8001BS | BS | Tengger Desert |
| SPT8002BS | BS | Tengger Desert |
| SPT8003LC | LC | Tengger Desert |
| SPT8004LC | LC | Tengger Desert |
| SPT8005CC | CC | Tengger Desert |
| SPT8006CC | CC | Tengger Desert |
| SPT8007MC | MC | Tengger Desert |
| SPT8008MC | MC | Tengger Desert |
| BD201610S1 | NV | Badain Jaran Desert |
| BD201610S2 | NV | Badain Jaran Desert |
| BD201610S3 | NV | Badain Jaran Desert |
| BD201610S4 | SL | Badain Jaran Desert |
| BD201610S5 | SL | Badain Jaran Desert |
| BD201610S6 | SP | Badain Jaran Desert |
| BD201610S7 | SP | Badain Jaran Desert |

**Table S4.** The metastats analysis of Badain Jaran Desert and Tengger Desert

|  | **group1**  **(BD201610S1,BD201610S2,BD201610S3,BD201610S4,BD201610S5,BD201610S6,BD201610S7)** | | | **group2**  **(SPT8001BS,SPT8002BS,SPT8003LC,SPT8004LC,SPT8005CC,SPT8006CC,SPT8007MC,SPT8008MC)** | | | |  |  |  |
| --- | --- | --- | --- | --- | --- | --- | --- | --- | --- | --- |
| feature | mean | variance | standard | mean | variance | standard | p value | | q value |  |
| Aciditerrimonas | 0.0003566 | 2.57E-07 | 0.000192 | 0.000738 | 6.90E-07 | 0.0002936 | | 1 | 1 | |
| Actinomadura | 0 | 0 | 0 | 0.000132 | 1.39E-07 | 0.0001317 | | 1 | 1 | |
| Actinomyces | 6.67E-05 | 3.12E-08 | 6.67E-05 | 0 | 0 | 0 | | 1 | 1 | |
| Actinomycetospora | 0.0029909 | 8.88E-06 | 0.001126 | 0.001909 | 3.09E-06 | 0.0006211 | | 1 | 1 | |
| Actinophytocola | 0.0088992 | 0.00021 | 0.005471 | 0.00927 | 0.000403 | 0.0070933 | | 1 | 1 | |
| Actinoplanes | 0.0047762 | 3.44E-05 | 0.002216 | 0.053837 | 0.003505 | 0.02093 | | 1 | 1 | |
| Actinotalea | 6.67E-05 | 3.12E-08 | 6.67E-05 | 0 | 0 | 0 | | 1 | 1 | |
| Aeromicrobium | 0.0026803 | 5.82E-06 | 0.000912 | 0.007936 | 0.000331 | 0.0064352 | | 1 | 1 | |
| Agrococcus | 0.0004007 | 1.12E-06 | 0.000401 | 0 | 0 | 0 | | 1 | 1 | |
| Agromyces | 0.0004204 | 3.41E-07 | 0.000221 | 0.001903 | 2.19E-05 | 0.0016558 | | 1 | 1 | |
| Amycolatopsis | 5.05E-05 | 1.78E-08 | 5.05E-05 | 4.41E-05 | 1.56E-08 | 4.41E-05 | | 1 | 1 | |
| Angustibacter | 0 | 0 | 0 | 0.000391 | 1.22E-06 | 0.0003909 | | 1 | 1 | |
| Aquipuribacter | 0.0001335 | 1.25E-07 | 0.000133 | 0 | 0 | 0 | | 1 | 1 | |
| Arthrobacter | 0.2392219 | 0.008119 | 0.034056 | 0.09209 | 0.007852 | 0.0313294 | | 1 | 1 | |
| Atopobium | 0.0014836 | 9.57E-06 | 0.001169 | 0 | 0 | 0 | | 1 | 1 | |
| Bifidobacterium | 0.0039658 | 0.000101 | 0.003792 | 0 | 0 | 0 | | 1 | 1 | |
| Blastococcus | 0.1282301 | 0.008866 | 0.035588 | 0.101718 | 0.008592 | 0.0327724 | | 1 | 1 | |
| Brachybacterium | 0.0037496 | 7.59E-05 | 0.003293 | 0.002957 | 6.87E-05 | 0.0029308 | | 1 | 1 | |
| Aquiluna | 0 | 0 | 0 | 9.72E-05 | 2.03E-08 | 5.04E-05 | | 1 | 1 | |
| Microthrix | 0.0003923 | 1.08E-06 | 0.000392 | 2.08E-05 | 3.45E-09 | 2.08E-05 | | 1 | 1 | |
| Planktophila | 0 | 0 | 0 | 2.11E-05 | 3.56E-09 | 2.11E-05 | | 1 | 1 | |
| Cellulomonas | 0.0263364 | 0.000361 | 0.007181 | 0.021435 | 0.001386 | 0.0131646 | | 1 | 1 | |
| Clavibacter | 0.0006011 | 2.53E-06 | 0.000601 | 0 | 0 | 0 | | 1 | 1 | |
| Conexibacter | 0.000602 | 2.03E-06 | 0.000538 | 4.41E-05 | 1.56E-08 | 4.41E-05 | | 1 | 1 | |
| Corynebacterium | 0.0014885 | 1.27E-05 | 0.001347 | 0 | 0 | 0 | | 1 | 1 | |
| Crossiella | 0.0010415 | 3.77E-06 | 0.000734 | 0.014894 | 0.000674 | 0.0091775 | | 1 | 1 | |
| Cryptosporangium | 0.0016728 | 7.86E-06 | 0.00106 | 0.001127 | 1.81E-06 | 0.0004759 | | 1 | 1 | |
| Dactylosporangium | 0.0002425 | 1.83E-07 | 0.000162 | 0.002908 | 2.25E-05 | 0.0016771 | | 1 | 1 | |
| Demequina | 0.0001335 | 1.25E-07 | 0.000133 | 0 | 0 | 0 | | 1 | 1 | |
| Euzebya | 0.0338082 | 0.003348 | 0.021871 | 0.007534 | 9.51E-05 | 0.0034473 | | 1 | 1 | |
| Fodinicola | 0.0001961 | 2.69E-07 | 0.000196 | 0 | 0 | 0 | | 1 | 1 | |
| Frankia | 0.0004822 | 4.60E-07 | 0.000256 | 0.015424 | 0.001833 | 0.0151353 | | 1 | 1 | |
| Friedmanniella | 0.0045206 | 2.49E-05 | 0.001888 | 0.016074 | 0.000147 | 0.0042857 | | 1 | 1 | |
| Frigoribacterium | 0.0005343 | 2.00E-06 | 0.000534 | 0 | 0 | 0 | | 1 | 1 | |
| Gaiella | 0.0019515 | 1.04E-05 | 0.001219 | 0.009887 | 0.000423 | 0.0072697 | | 1 | 1 | |
| Geodermatophilus | 0.058013 | 0.002722 | 0.01972 | 0.046445 | 0.006468 | 0.028434 | | 1 | 1 | |
| Georgenia | 0.0003641 | 3.16E-07 | 0.000212 | 0.000187 | 4.68E-08 | 7.65E-05 | | 1 | 1 | |
| Glycomyces | 3.17E-05 | 7.04E-09 | 3.17E-05 | 0.000353 | 9.98E-07 | 0.0003532 | | 1 | 1 | |
| Gordonia | 0.0001871 | 1.71E-07 | 0.000156 | 0 | 0 | 0 | | 1 | 1 | |
| Haloactinopolyspora | 0 | 0 | 0 | 0.000812 | 4.11E-06 | 0.000717 | | 1 | 1 | |
| Iamia | 0.0026192 | 1.07E-05 | 0.001239 | 0.005584 | 2.42E-05 | 0.0017376 | | 1 | 1 | |
| Illumatobacter | 0.0035323 | 8.73E-05 | 0.003532 | 0 | 0 | 0 | | 1 | 1 | |
| Janibacter | 2.53E-05 | 4.47E-09 | 2.53E-05 | 0.002622 | 5.28E-05 | 0.0025699 | | 1 | 1 | |
| Kibdelosporangium | 0.0001362 | 1.30E-07 | 0.000136 | 0.006334 | 0.000301 | 0.0061321 | | 1 | 1 | |
| Kineococcus | 0.0055451 | 0.000148 | 0.004596 | 0.004331 | 0.000132 | 0.0040615 | | 1 | 1 | |
| Kineosporia | 0.0047244 | 8.12E-05 | 0.003406 | 0.003454 | 7.60E-05 | 0.0030825 | | 1 | 1 | |
| Kocuria | 0.0908382 | 0.006163 | 0.029671 | 0.033015 | 0.004569 | 0.0238984 | | 1 | 1 | |
| Krasilnikovia | 0 | 0 | 0 | 0.000797 | 3.39E-06 | 0.0006513 | | 1 | 1 | |
| Kribbella | 0.0021369 | 2.82E-06 | 0.000635 | 0.016264 | 0.000375 | 0.0068498 | | 1 | 1 | |
| Lentzea | 0.0024069 | 2.51E-05 | 0.001894 | 0.005788 | 5.67E-05 | 0.0026621 | | 1 | 1 | |
| Longispora | 0.0006357 | 2.83E-06 | 0.000636 | 0.001102 | 9.71E-06 | 0.0011015 | | 1 | 1 | |
| Lysinimonas | 0.0005611 | 6.56E-07 | 0.000306 | 0.00095 | 2.56E-06 | 0.0005661 | | 1 | 1 | |
| Marisediminicola | 0.0002672 | 5.00E-07 | 0.000267 | 0 | 0 | 0 | | 1 | 1 | |
| Marmoricola | 0.0043979 | 2.45E-05 | 0.00187 | 0.01275 | 8.63E-05 | 0.0032852 | | 1 | 1 | |
| Microbacterium | 0.0063747 | 0.000104 | 0.003856 | 0.001072 | 4.30E-06 | 0.0007334 | | 1 | 1 | |
| Microbispora | 0 | 0 | 0 | 6.58E-05 | 3.47E-08 | 6.58E-05 | | 1 | 1 | |
| Microlunatus | 9.71E-05 | 1.50E-08 | 4.62E-05 | 0.002572 | 3.61E-06 | 0.0006718 | | 1 | 1 | |
| Micromonospora | 0.0138877 | 0.000849 | 0.011012 | 0.00539 | 7.05E-06 | 0.0009385 | | 1 | 1 | |
| Modestobacter | 0.0647388 | 0.006491 | 0.030451 | 0.042917 | 0.002077 | 0.0161135 | | 1 | 1 | |
| Motilibacter | 0.0003959 | 4.57E-07 | 0.000256 | 0 | 0 | 0 | | 1 | 1 | |
| Mycetocola | 0.0001335 | 1.25E-07 | 0.000133 | 0 | 0 | 0 | | 1 | 1 | |
| Mycobacterium | 0.0015094 | 3.63E-06 | 0.00072 | 0.005024 | 6.16E-05 | 0.0027739 | | 1 | 1 | |
| Nakamurella | 0.0003608 | 3.20E-07 | 0.000214 | 3.08E-05 | 7.58E-09 | 3.08E-05 | | 1 | 1 | |
| Nesterenkonia | 0.0051426 | 0.000185 | 0.005143 | 0 | 0 | 0 | | 1 | 1 | |
| Nitriliruptor | 0.054126 | 0.008562 | 0.034974 | 0.000843 | 1.54E-06 | 0.0004388 | | 1 | 1 | |
| Nocardioides | 0.0470356 | 0.00375 | 0.023145 | 0.04129 | 0.00152 | 0.0137826 | | 1 | 1 | |
| Nocardiopsis | 0.0001105 | 8.55E-08 | 0.000111 | 0.000117 | 7.37E-08 | 9.60E-05 | | 1 | 1 | |
| Ornithinicoccus | 0.0001268 | 1.13E-07 | 0.000127 | 0 | 0 | 0 | | 1 | 1 | |
| Ornithinimicrobium | 0.0007697 | 1.71E-06 | 0.000494 | 0.000723 | 1.68E-06 | 0.0004582 | | 1 | 1 | |
| Other | 0.0059161 | 3.84E-05 | 0.002341 | 0.017378 | 0.000136 | 0.0041167 | | 1 | 1 | |
| Patulibacter | 0.0080739 | 2.93E-05 | 0.002044 | 0.033208 | 0.000934 | 0.0108046 | | 1 | 1 | |
| Promicromonospora | 0.000804 | 1.10E-06 | 0.000396 | 0.001544 | 1.22E-05 | 0.0012342 | | 1 | 1 | |
| Propionibacterium | 0.0034376 | 4.99E-05 | 0.00267 | 0 | 0 | 0 | | 1 | 1 | |
| Pseudonocardia | 0.0119568 | 0.000303 | 0.006584 | 0.04106 | 0.002792 | 0.0186828 | | 1 | 1 | |
| Quadrisphaera | 7.37E-05 | 3.80E-08 | 7.37E-05 | 0.000792 | 1.49E-06 | 0.0004318 | | 1 | 1 | |
| Rhodococcus | 0 | 0 | 0 | 2.08E-05 | 3.45E-09 | 2.08E-05 | | 1 | 1 | |
| Rothia | 0.0003179 | 7.07E-07 | 0.000318 | 0 | 0 | 0 | | 1 | 1 | |
| Rubrobacter | 0.0497598 | 0.000733 | 0.010231 | 0.164083 | 0.020978 | 0.0512079 | | 1 | 1 | |
| Saccharothrix | 0.0024666 | 1.51E-05 | 0.001471 | 0.017814 | 0.000692 | 0.0093005 | | 1 | 1 | |
| Solirubrobacter | 0.0283952 | 0.001625 | 0.015237 | 0.025893 | 0.000322 | 0.0063454 | | 1 | 1 | |
| Sporichthya | 0.0003801 | 2.27E-07 | 0.00018 | 0.000865 | 1.14E-06 | 0.0003768 | | 1 | 1 | |
| Streptomyces | 0.0153675 | 0.000152 | 0.004666 | 0.011944 | 0.000622 | 0.0088202 | | 1 | 1 | |
| Umezawaea | 0.0001262 | 1.12E-07 | 0.000126 | 0.000455 | 1.05E-06 | 0.000362 | | 1 | 1 | |
| Verrucosispora | 0.0255413 | 0.000862 | 0.011094 | 0.070082 | 0.004156 | 0.0227923 | | 1 | 1 | |
| Virgisporangium | 0.0016291 | 1.43E-05 | 0.001431 | 0.008814 | 0.000418 | 0.00723 | | 1 | 1 | |
| Williamsia | 0.0003805 | 1.01E-06 | 0.000381 | 0 | 0 | 0 | | 1 | 1 | |
| Yonghaparkia | 0.0026461 | 4.89E-06 | 0.000836 | 0.00282 | 2.00E-05 | 0.0015824 | | 1 | 1 | |
