# Supplementary material for: Diversity of Bacteria and the Characteristics of Actinobacteria Community Structure in Badain Jaran Desert and Tengger Desert of China
Source: Front Microbiol. 2018 May 23;9:1068. doi: 10.3389/fmicb.2018.01068 (PMC5974926; doi:10.3389/fmicb.2018.01068)
Supplement: Supplementary file 2 [file Data_Sheet_2.docx]

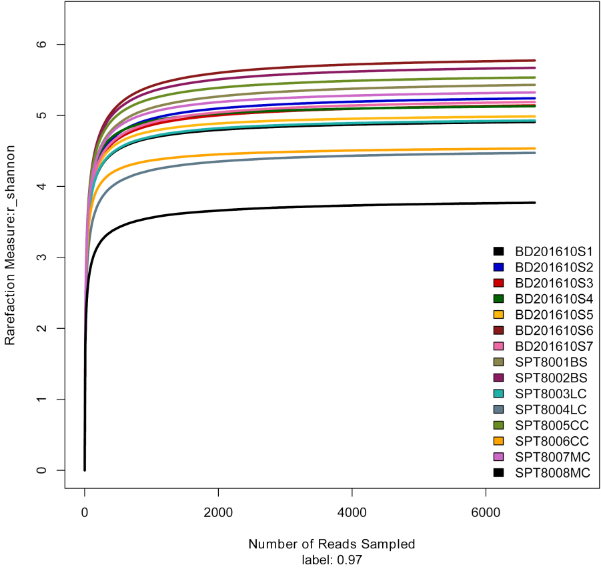


**FIGURE S1 | The Shannon index curves of 15 sand samples.**

**
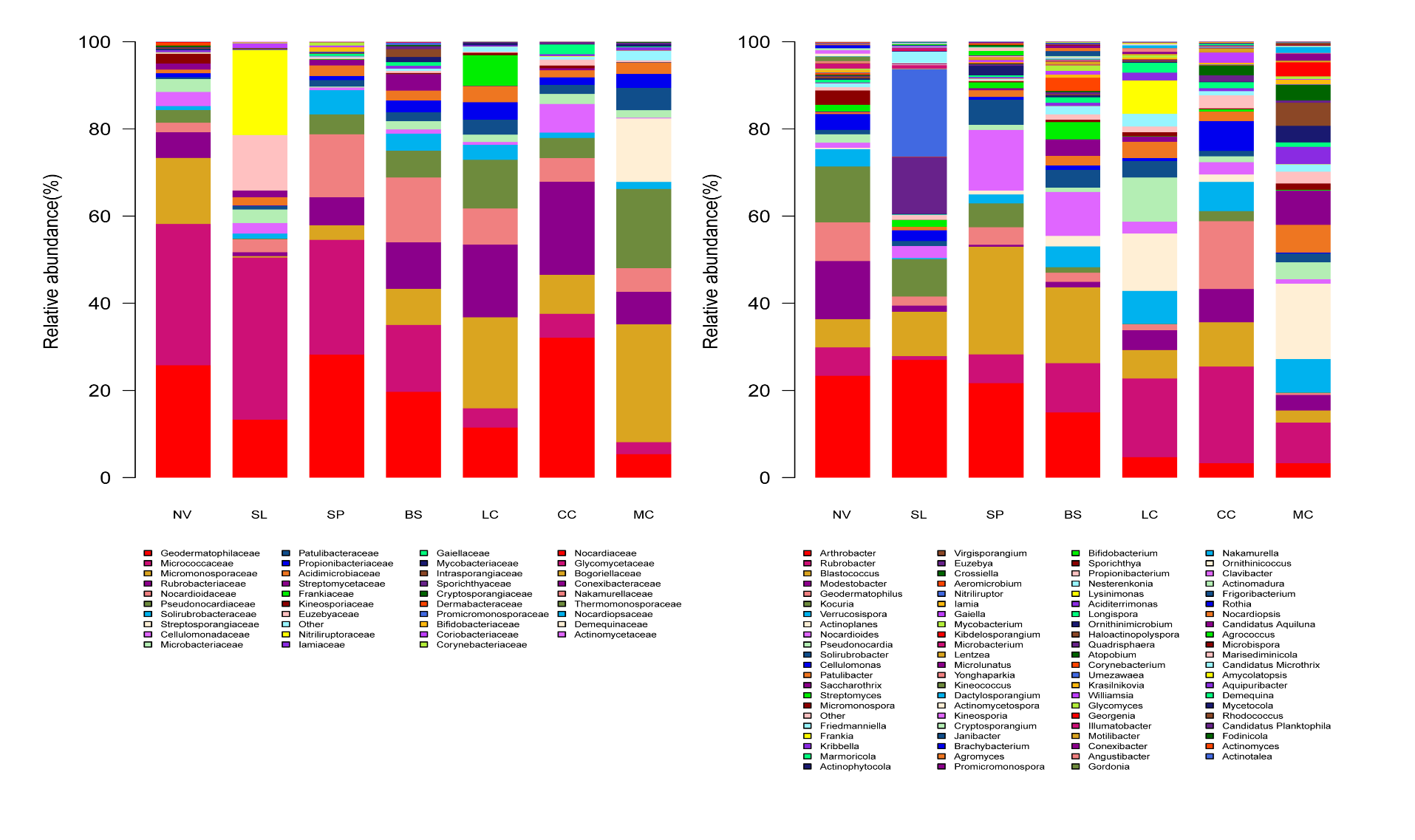
**

**(B)**

**(A)**

**FIGURE S2 | The relative abundances of different actinobacterial taxon level in different types of the two deserts in HTS method.** (A) Family taxon level; (B) Genus taxon level.

A

**(B)**

**(A)**

**FIGURE S3 | Dendrogram based on 16S rRNA gene sequences analysis of actinobacterial isolates from the two deserts. (A) Badain Jaran Desert; (B) Tengger Desert.** Bar, 0.01 and 0.005 substitutions per nucleotide position in A and B, respectively.


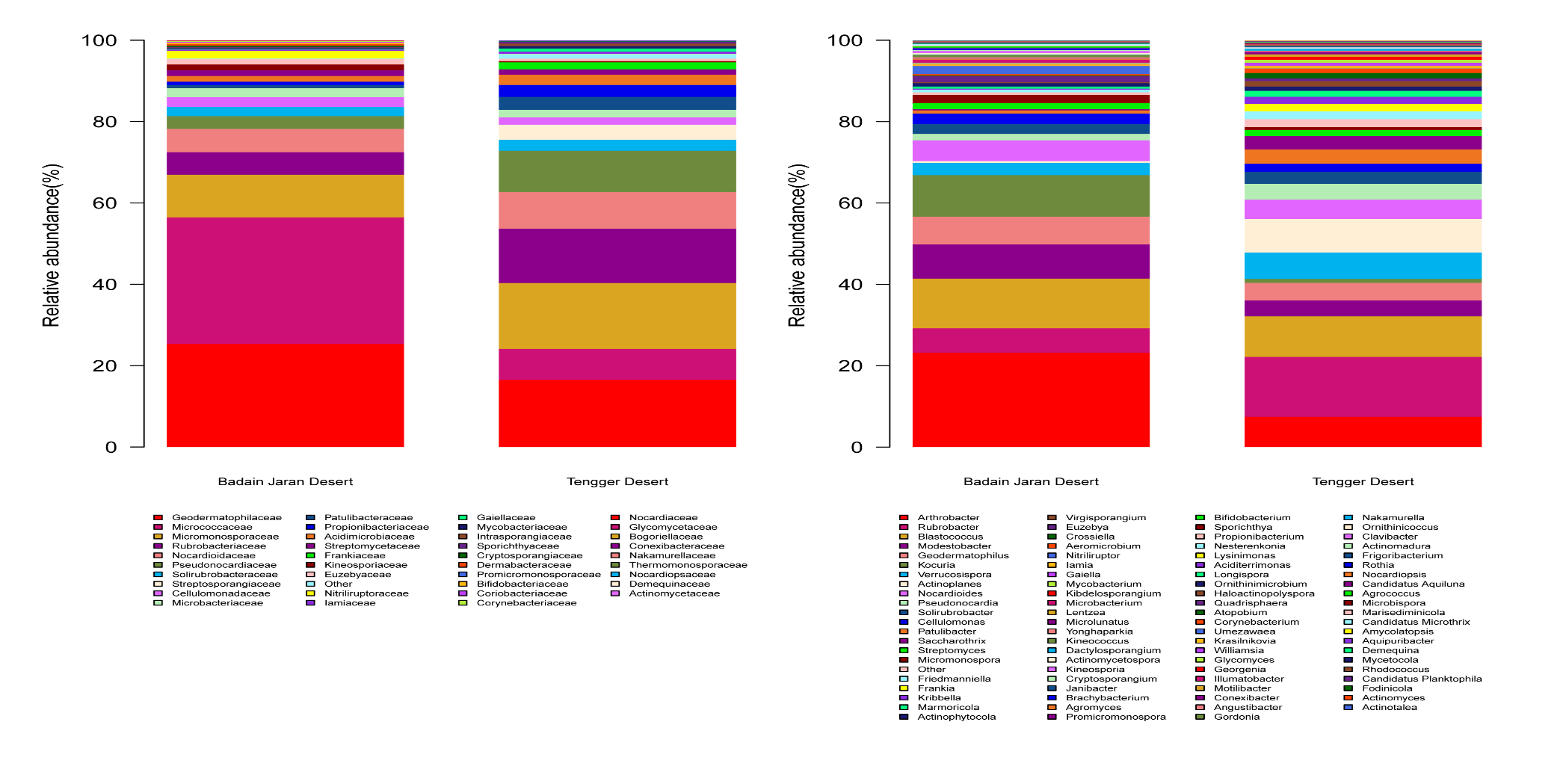
 **FIGURE S4 | The relative abundances of different actinobacterial taxon level of the two deserts in HTS method.** (A)Family taxon level; (B) Genus taxon level.

**(B)**

**(A)**
